# Geographical Variation of Antimicrobial Resistance of *Salmonella* in Japanese Chicken

**DOI:** 10.14252/foodsafetyfscj.D-24-00002

**Published:** 2024-08-30

**Authors:** Yoshimasa Sasaki, Tetsuya Ikeda, Yoshika Momose, Kenzo Yonemitsu, Masashi Uema, Tetsuo Asai

**Affiliations:** 1 National Institute of Health Sciences, 3-25-26, Tonomachi, Kawasaki-ku, Kawasaki, Kanagawa 210-9501, Japan; 2 Division of Veterinary Science, Department of Veterinary Medicine, Obihiro University of Agriculture and Veterinary Medicine, Inada-cho, Obihiro, Hokkaido 080-8555, Japan; 3 Department of Infectious Diseases, Hokkaido Institute of Public Health, Kita19 Nishi 12, Kita-ku Sapporo, Hokkaido 060-0819, Japan; 4 Murayama Branch, National Institute of Infectious Diseases. 4-7-1, Gakuen, Musashimurayama, Tokyo 208-0011, Japan; 5 Department of Applied Veterinary Science, the United Graduate School of Veterinary Science, Gifu University, 1-1, Yanagido, Gifu 501-1193, Japan

**Keywords:** Antimicrobial resistance, Chicken meat, *Salmonella*

## Abstract

Chicken is a potent source of *Salmonella* infection in humans. Occasionally, patients with severe *Salmonella* enteritis require antimicrobial therapy. Antimicrobials are used to prevent and treat bacterial infections in broiler and breeder farms. Herein, we investigated the prevalence and antimicrobial resistance of *Salmonella* in 337 vacuum-packed chicken breast products manufactured in Japan between June and December 2021. *Salmonella* was isolated from 287 samples (85.2%). Among the products from Eastern Japan, the lowest *Salmonella* prevalence was observed in those processed in September (65.6%), which was significantly (*p* < 0.05) lower than that in November or December. Among the products from Western Japan, the lowest *Salmonella* prevalence was observed in those processed in August (61.9%), which was significantly (*p* < 0.05) lower than that in June, November, and December. The most frequent serovar was *Salmonella* Schwarzengrund (223 isolates), followed by *S.* Infantis (53 isolates), *S.* Manhattan (9 isolates), and *S.* Enteritidis (1 isolate). High rates of antimicrobial resistance were observed for streptomycin (64.5%), kanamycin (50.2%), tetracycline (65.2%), nalidixic acid (11.5%), and trimethoprim (35.9%). Resistance rates against these five antimicrobials in *S*. Schwarzengrund isolates were markedly higher in the isolates from Western Japan than in those from Eastern Japan. All 287 *Salmonella* isolates were susceptible to ciprofloxacin which belongs to fluoroquinolones and cefotaxime which belongs to third-generation cephalosporins. *Salmonella* prevalence in chicken products in Japan was found to be extremely high; therefore, chicken meat should be thoroughly heated before consumption. In Japan, fluoroquinolones and third-generation cephalosporins are recommended as the first- and second-choice antimicrobials for patients with severe *Salmonella* enteritis, respectively. The results of this study show that administering fluoroquinolones or third-generation cephalosporins is an effective option for patients with *Salmonella* enteritis caused by consuming chicken meat, and efficient strategies for *Salmonella* management on broiler farms and chicken-processing plants need to be developed.

## 1. Introduction

*Salmonella* is a bacterial pathogen that causes foodborne illnesses. The World Health Organization states that non-typhoidal *Salmonella* is one of the four key global causative agents of diarrhea^1^^)^. *Salmonella* is classified into >2,600 serotypes based on three structures: somatic (O), flagellar, and capsular surface antigens. Four O-serogroups (O:4, O:7, O:8, and O:9) are the major contributors to human non-typhoidal salmonellosis^2^^,^^3^^,^^4^^,^^5^^)^. The top five frequent *Salmonella* serotypes in patients in 2020 following the Infectious Agents Surveillance Report (https://kansen-levelmap.mhlw.go.jp/Byogentai/Pdf/data81j.pdf) were *S*. Thompson (O:7), *S*. Enteritidis (O:9), *S.* Schwarzengrund (O:4), *S*. Braenderup (O:7), and *S*. Typhimurium (O:4). Although the annual number of food poisoning outbreaks in Japan caused by *Salmonella* spp. has been <50 since 2011, the average number of patients per *Salmonella* outbreak is 38, which is approximately five times the number of patients per *Campylobacter* outbreak^6^^)^.

Chicken meat is a major cause of foodborne salmonellosis in Japan^7^^)^. Human nontyphoidal salmonellosis typically causes acute self-limiting enteritis. Antimicrobial therapy may be lifesaving in patients with severe symptoms and health risk groups such as infants, the elderly, and immunocompromised patients although it is not recommended in various cases^1^^,^^8^^)^. Fluoroquinolones, such as ciprofloxacin and levofloxacin, and third-generation cephalosporins (TGCs), such as cefotaxime and ceftriaxone, have been classified as “critically important antimicrobials for human medicine” by the World Health Organization^9^^)^. Antimicrobials such as penicillins, tetracyclines, aminoglycosides, fluoroqinolones, sulfonamides, and macrolides, are used to treat bacterial infections in broilers in Japan^10^^)^. Moreover, some of them are routinely used in breeding farms and hatcheries to prevent and treat bacterial infections^11^^,^^12^^)^. Numerous Japanese studies have reported multidrug-resistant *Salmonella* isolated from broilers^10^^,^^11^^,^^13^^,^^14^^)^. thus, by consuming chicken meat, humans can be infected with multidrug-resistant *Salmonella*. Therefore, antimicrobial resistance of *Salmonella* isolated from chicken meat is an important issue in chemotherapeutically treating patients with enteritis. Additionally, there are no studies on geographical variation in the prevalence and antimicrobial resistance of *Salmonella* in chicken meat in Japan. This study aimed to determine the antimicrobial resistance profiles of *Salmonella* isolated from chicken meat. The results of this study may help characterize *Salmonella* isolated from chicken meat and select antimicrobials for treating patients with enteritis.

## 2. Materials and Methods

### 2.1 Sampling

A total of 337 chilled chicken breast products consisting of three–six breast pieces vacuum-packed at local processing plants were collected from retail shops in six prefectures (Hokkaido, Saitama, Chiba, Tokyo, Kanagawa, and Kagoshima) from June to December 2021. The product label indicated the name and address of the processing plant and the production lot number. Samples were collected from each production lot. The refrigerated products were sent to the National Institute of Health Sciences via express delivery and stored in the laboratory at 4°C until examination. The samples were examined within 48 h of collection.

### 2.2 *Salmonella* isolation

Each sample consisted of the skin (75 g) taken from more than three meat pieces placed in a plastic bag containing 75 mL of buffered peptone water (BPW; Oxoid Ltd., Hampshire, UK). After stomaching, 50 mL of the solution was mixed with 200 mL of BPW and incubated at 37°C for 18 h for pre-enrichment. After incubation, 0.1 and 1 mL of the culture were added to 10 mL of Rappaport–Vassiliadis broth (Oxoid) and 10 mL of Hajna tetrathionate broth (Eiken Chemical, Tokyo, Japan), respectively, and were subsequently incubated at 42°C for 20 h. After incubation, each culture was streaked onto two selective isolation agar plates: xylose–lysine–deoxycholate agar (Oxoid) and CHROMagar^™^*Salmonella* (CHROMagar, Paris, France) and subsequently incubated at 37°C for 24 h. A maximum of four suspected *Salmonella* isolates per sample were biochemically identified, as previously described^15^^)^. The isolates were tested for slide agglutination using O antisera (Denka Co., Tokyo, Japan) and tube agglutination using H antisera (Denka). The serovars were determined based on their reaction with O- and H-group antigens according to the Kauffmann–White scheme^16^^)^. One serovar per sample was suspended in 20% glycerol and stored at −80°C until ready for antimicrobial susceptibility testing.

### 2.3 Antimicrobial Susceptibility Testing of the *Salmonella* isolates

Following broth microdilution on dried plates (Eiken Chemical, Tokyo, Japan), isolate susceptibility to ampicillin (1–128 mg/L), cefazolin (1–128 mg/L), cefotaxime (0.5–64 mg/L), streptomycin (1–128 mg/L), gentamicin (0.5–64 mg/L), kanamycin (1–128 mg/L), tetracycline (0.5–64 mg/L), nalidixic acid (1–128 mg/L), ciprofloxacin (0.03–4 mg/L), colistin (0.12–16 mg/L), chloramphenicol (1–128 mg/L), and trimethoprim (0.25–16 mg/L) was tested. *Escherichia coli* ATCC 25922 was used as the quality control strain. The breakpoints for ampicillin (32 mg/L), cefazolin (8 mg/L), cefotaxime (4 mg/L), streptomycin (32 mg/L), gentamicin (16 mg/L), kanamycin (64 mg/L), tetracycline (16 mg/L), nalidixic acid (32 mg/L), ciprofloxacin (1 mg/L), colistin (4 mg/L), chloramphenicol (32 mg/L), and trimethoprim (16 mg/L) were adopted from the Clinical and Laboratory Standards Institute^17^^)^ and the Japanese Veterinary Antimicrobial Resistance Monitoring System^10^^)^.

### 2.4 Statistical Analyses

All statistical analyses were performed using R version 4.1. The differences between the proportions were tested using Fisher’s exact test, where *p*-values < 0.05 were considered statistically significant.

### 2.5 Investigation into the Use of Antimicrobials at Chicken Processing Plants with Breeder Farms and Hatcheries

We collected chicken breast products from 11 chicken processing plants that owned breeder farms. We enquired whether they routinely used antimicrobials to prevent bacterial infections among their breeder farms and hatcheries. Furthermore, we asked them to share the names of antimicrobials either during the interview or via e-mail.

## 3. Results

A total of 337 vacuum-packed products from 21 chicken-processing plants (A to U) were collected from 26 retail shops. Among the 21 plants, 12 and 9 were located in Eastern Japan (Hokkaido, Aomori, Iwate, Miyagi, Gunma, and Chiba prefectures) and Western Japan (Tokushima, Saga, Miyazaki, and Kagoshima prefectures), respectively. *Salmonella* spp. were isolated from 287 (85.2%) products packaged in 20 (95.2%) plants (Table 1). Among the products processed in Eastern Japan, the lowest prevalence of *Salmonella* was observed in September (65.6%). *Salmonella* prevalence was significantly (*p* < 0.05) lower in September than in November or December. Among the products processed in Western Japan, the lowest *Salmonella* prevalence was observed in August (61.9%). *Salmonella* prevalence was significantly (*p* < 0.05) lower in August than in June, November, and December.

**Table 1.**
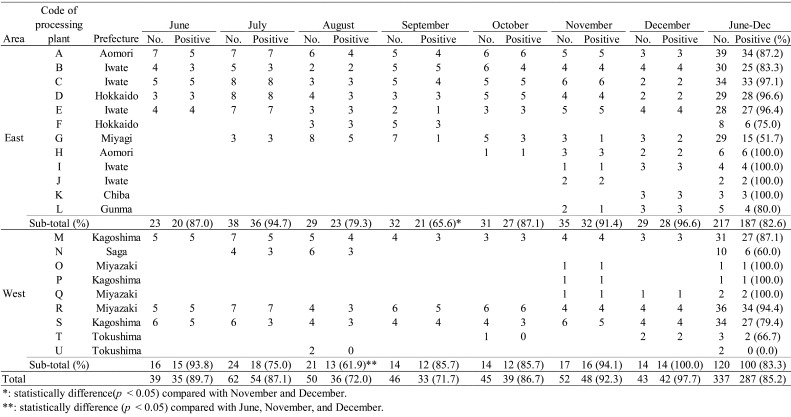
Monthly *Salmonella* prevalence in the breast meat.

*Salmonella* isolates obtained from 187 *Salmonella*-positive products processed in Eastern Japan were serotyped into four serovars. One *Salmonella* serovar was isolated from one *Salmonella*-positive product. The most frequently detected serovar was *S*. Schwarzengrund (124 products), followed by *S*. Infantis (53 products), *S*. Manhattan (9 products), and *S*. Enteritidis (1 product) (Table 2). Products contaminated with *S*. Schwarzengrund and *S*. Infantis were processed in 11 plants situated in all the prefectures investigated other than Gunma prefecture and in seven plants situated in all the prefectures investigated, respectively. Products contaminated with *S*. Manhattan were processed at a plant located in Hokkaido. Among the 100 *Salmonella*-positive products processed in Western Japan, although one isolate obtained from one product was untypeable, all isolates obtained from the remaining 99 products were *S*. Schwarzengrund. One *Salmonella* serovar was isolated from one *Salmonella*-positive product. Products contaminated with *S*. Schwarzengrund were processed from eight plants in all the investigated prefectures.

**Table 2.**
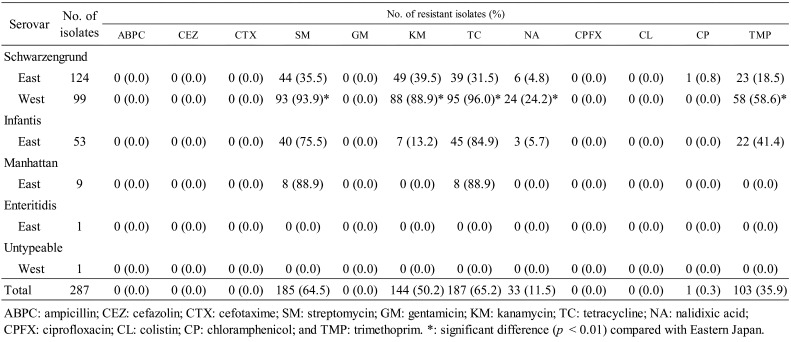
Antimicrobial resistance of *Salmonella* isolated from the breast meat products.

The resistance rates to streptomycin, kanamycin, tetracycline, nalidixic acid, chloramphenicol, and trimethoprim were 64.5%, 50.2%, 65.2%, 11.5%, 0.3%, and 35.9%, respectively. All isolates were susceptible to ampicillin, cefazolin, cefotaxime, gentamicin, ciprofloxacin, and colistin. Among the *S*. Schwarzengrund isolates, the rates of resistance to streptomycin, kanamycin, tetracycline, nalidixic acid, and trimethoprim were significantly (*p* < 0.01) lower in Eastern Japan than in Western Japan. The highest resistance rate for the *S*. Infantis isolates was observed for tetracycline (84.9%), followed by streptomycin (75.5%), trimethoprim (41.4%), and kanamycin (13.2%). The resistance rates among the *S*. Manhattan isolates against both streptomycin and tetracycline were 88.9% and no isolates were resistant to the other tested antimicrobials.

Relationships existed between the processing plants where the products were processed and the *S*. Schwarzengrund’s antimicrobial resistance. In Eastern Japan, the kanamycin resistance rate (91.3%, 21/23) in plants that did not own breeder farms was higher than that in isolates from plants that owned breeder farms (27.7%, 28/101) (Table 3). In Western Japan, the nalidixic acid resistance rate (42.6%, 20/47) in plants that did not own breeder farms was higher than that in isolates from plants that owned breeder farms (7.7%, 4/52). We asked processing plants that owned breeder farms whether they routinely used antimicrobials to prevent bacterial infections on their breeder farms and hatcheries. Eight plants (A to D in Eastern Japan and M to P in Western Japan) responded to this question on the condition of anonymity. Of the four processing plants (A to D) situated in Eastern Japan, three (A, B, and D) did not use antimicrobials to prevent bacterial infections among their breeder farms or hatcheries. The remaining plant (C) used oxytetracycline on its breeder farms. The four processing plants (M to P) in Western Japan used antimicrobials to prevent bacterial infections in breeder farms and hatcheries. Tetracycline resistance (79.5%, 58/73) in *S*. Schwarzengrund from five processing plants (C, M, N, O, and P) that used oxytetracycline in their breeder farms was significantly (*p* < 0.01) higher than that (27.7%, 18/65) from three processing plants (A, B, and D) that did not use it in their breeder farms. Kanamycin resistance (89.8%, 44/49) in *S*. Schwarzengrund originating from three processing plants (M, N, and O) that used kanamycin in their hatcheries was significantly (*p* < 0.01) higher than that (31.5%, 28/89) in *S*. Schwarzengrund originating from five processing plants (A, B, C, D, and P) that did not use it in their hatcheries.

**Table 3.**
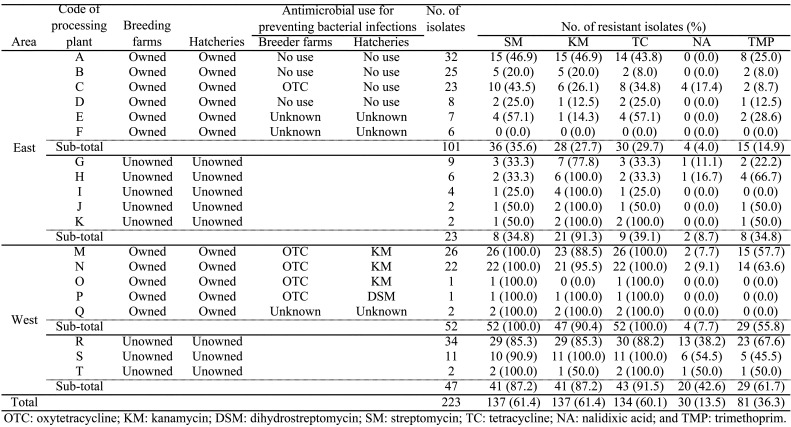
Antimicrobial used in breeding farms and hatcheries and antimicrobial resistance in isolated *S* . Schwarzengrund.

## 4. Discussion

Herein, we collected local chicken breast products that were vacuum-packed at chicken processing plants to eliminate the possibility of *Salmonella* contamination after shipment. Chicken breast products were packaged in 21 chicken processing plants located in 10 prefectures. According to the Statistical Survey on Livestock of the Japanese Ministry of Agriculture, Forestry, and Fisheries (JMAFF) (https://www.maff.go.jp/j/tokei/kouhyou/tikusan/index.html), these 10 prefectures produced approximately 75.8% of the total number of broilers in Japan in 2021. The *Salmonella* prevalence in broiler flocks in Japan is >80%. For instance, in a national survey conducted between 2007 and 2008, *Salmonella* prevalence in broiler flocks was 86%^14^^)^. Hence, this survey was conducted with the expectation that *Salmonella* prevalence in chicken meat would be extremely high. The average *Salmonella* prevalence during the investigation period was 85.2%, and no difference in *Salmonella* prevalence was observed between Eastern and Western Japan. This finding was consistent with our expectations. Furthermore, although it was not possible to purchase the same number of products processed at each chicken processing plants each month, *Salmonella* prevalence in products processed in Eastern and Western Japan ranged from 65.6% (September) to 96.6% (December) and from 61.9% (August) to 100% (December), respectively. *S*. Schwarzengrund contamination of chicken meat was previously more frequently detected during spring and winter than during summer^18^^)^. Recently, we collected cecal contents and breast meat products from 35 broiler flocks at six chicken processing plants in Kyushu, Japan between June and October 2022, and found that the lowest *Salmonella* prevalence for both cecal contents and chicken meat products was in August^19^^)^. The most common serovar in this report was also *S*. Schwarzengrund. In the present study, *S*. Schwarzengrund was the most frequent serovar, accounting for 81.2% of the *Salmonella* isolates. The seasonal variability in *Salmonella* prevalence observed may be shaped by *S*. Schwarzengrund. Although the European Union-wide baseline survey of *Salmonella* in broiler flocks demonstrated that *Salmonella* prevalence was higher in winter than in summer, no nationwide survey of broiler flocks throughout the year has been conducted in Japan^20^^)^. To understand the seasonality of *Salmonell*a prevalence in chicken products, investigating *Salmonella* prevalence in broiler flocks throughout the year is necessary.

Although the JMAFF has not approved the use of antimicrobials to prevent bacterial infections in livestock, antimicrobials have been used for several years in the poultry industry to treat and prevent bacterial infections. For instance, dihydrostreptomycin is routinely added to vaccines during *in ovo* vaccination at hatcheries^11^^)^. Additionally, oxytetracycline is routinely used to prevent mycoplasma infections in broiler breeder farms. Antimicrobial-resistant pathogenic bacteria arising from using antimicrobials in livestock can be transmitted to humans through livestock products, consequently reducing the efficacy of antimicrobial drugs in human chemical remedies. Public health concerns regarding antimicrobial resistance have increased over the years and using antimicrobials to prevent bacterial infections has gradually been discontinued. Ceftiofur, a TGC, was previously used in hatcheries; however, its use voluntarily ceased in March 2012 owing to increasing TGC resistance rates in *Escherichia coli* and *Salmonella* spp. isolated from broilers^10^^,^^14^^)^. After the withdrawal of ceftiofur, TGC resistance rates in *Salmonella* isolated from broiler and chicken meat decreased^21^^,^^22^^)^. In the present study, four processing plants (M to P) changed ceftiofur to kanamycin or dihydrostreptomycin after withdrawal and no TGC-resistant *Salmonella* was isolated. Furthermore, two processing plants (A and B) changed oxytetracycline to live mycoplasma vaccines for >10 years. Tetracycline resistance in *S*. Schwarzengrund from five processing plants (C, M, N, O, and P) that used oxytetracycline in their breeder farms was higher than that in *S*. Schwarzengrund originating from these two processing plants. Oxytetracycline withdrawal from breeder farms may have contributed to the reduced tetracycline resistance rate of *S*. Schwarzengrund in chicken meat.

Herein, the resistance rates to streptomycin, kanamycin, and tetracycline were >50%. High rates of resistance to kanamycin and tetracycline were associated with the use of kanamycin and oxytetracycline in hatcheries and breeder farms, respectively. In Japan, several day-old chick suppliers sell day-old chicks. Processing plants that do not own breeder farms purchase day-old chicks from some of them and raise chicks on their own or contracted broiler farms. Contrastingly, some large-scale processing plants purchase day-old chicks from day-old-chick suppliers and run their own breeder farms and hatcheries. Furthermore, they raise day-old chicks born at those hatcheries on their own or contracted farms. The high rates of kanamycin and tetracycline resistance in *S*. Schwarzengrund in Western Japan imply that the majority of breeder farms and hatcheries in Western Japan use oxytetracycline and kanamycin. Additionally, day-old chick suppliers in Western Japan may use quinolones in their breeder farms or hatcheries because nalidixic-acid resistance in *S*. Schwarzengrund from processing plants that did not own breeder farms was higher than that from processing plants that owned breeder farms. Contrastingly, in Eastern Japan, day-old chick suppliers may use kanamycin in their hatcheries because kanamycin resistance in *S*. Schwarzengrund from processing plants that did not own breeder farms was higher than that from processing plants that owned breeder farms. Day-old chick suppliers would routinely use antimicrobials in their breeder farms and hatcheries to ensure delivery of the day-old chick number requested by the processing plants to broiler farms. According to the JMAFF, the number of chicken farms exhibiting outbreaks of colibacillosis or staphylococcal infections is higher in Western Japan than in Eastern Japan (https://www.maff.go.jp/j/syouan/douei/kansi_densen/kanren_zyouhou.html) (*in Japanese*). Therefore, using antimicrobials to prevent bacterial infections may be more common in breeder farms and hatcheries in Western Japan than in Eastern Japan. Using antimicrobials in broiler farms may also have affected *Salmonella*’s antimicrobial resistance. Product labeling included the name of the processing plant, but not the name of the farm; therefore, we could not determine whether antimicrobials were used on the farm. Collecting samples from these facilities and obtaining information on antimicrobial use is necessary for clarifying the relationship between antimicrobial use and developing *Salmonella* drug resistance in breeder farms, hatcheries, and broiler farms.

*S*. Schwarzengrund was the most frequent serovar of *Salmonella* isolated from food workers, and the resistance rates to streptomycin, kanamycin, and tetracycline in this serovar were all >81%^23^^)^. Moreover, *S*. Schwarzengrund was recently reported to be the third most frequent serovar of *Salmonella* isolated from cases of human enteritis, and the resistance rates to streptomycin, kanamycin, and tetracycline in this serovar were all >38%^24^^)^. Contrastingly, *S*. Schwarzengrund is rarely isolated from retail pork or beef^25^^)^. Thus, antimicrobial-resistant *Salmonella* arising from using antimicrobials in breeder farms and hatcheries are transmitted to broiler farms and contaminated chicken meat. Eventually, humans will be infected with antimicrobial-resistant *Salmonella* by consuming contaminated chicken meat.

In conclusion, all investigated chicken processing plants distributed *Salmonella*-contaminated chicken products at high rates. Therefore, consumers should thoroughly cook chicken meat before consumption to prevent nontyphoidal salmonellosis. Moreover, efficient strategies for *Salmonella* management on broiler farms and chicken processing plants should be developed. In Japan, fluoroquinolones and TGCs are recommended as the first- and second-choice antimicrobials for patients with severe *Salmonella* enteritis, respectively^8^^)^. All *Salmonella* isolates were susceptible to ciprofloxacin and cefotaxime, although geographical variations in antimicrobial resistance of *Salmonella* in chicken products were clearly observed. Thus, administering fluoroquinolones or TGCs is an effective treatment option for patients with *Salmonella* enteritis caused by chicken meat consumption.
